# A chemometric approach to evaluate the effects of probe-type ultrasonication on the enzyme inactivation and quality attributes of fresh amla juice

**DOI:** 10.1016/j.ultsonch.2022.106268

**Published:** 2022-12-14

**Authors:** Raouf Aslam, Mohammed Shafiq Alam, Asgar Ali, Yang Tao, Sivakumar Manickam

**Affiliations:** a Department of Processing and Food Engineering, Punjab Agricultural University, Ludhiana-141 004, India; b Centre of Excellence for Postharvest Biotechnology (CEPB), School of Biosciences, University of Nottingham Malaysia, Jalan Broga, 43500 Semenyih, Selangor Darul Ehsan, Malaysia; c College of Food Science and Technology, Whole Grain Food Engineering Research Center, Nanjing Agricultural University, Nanjing 210095, Jiangsu, China; d Petroleum and Chemical Engineering, Faculty of Engineering, University Technology Brunei, Jalan Tungku Link Gadong, BE1410, Brunei Darussalam; e The UWA Institute of Agriculture, The University of Western Australia, Perth, WA 6001, Australia; f Leaders Institute, 76 Park Road, Woolloongabba, Queensland 4102, Australia

**Keywords:** Ultrasound, Cavitation, Enzymatic browning, *Emblica officinalis*, Response surface methodology

## Abstract

The enzymatic browning induced in amla juice due to the high activity of polyphenol oxidase (PPO) and peroxidase (POD) is one of the critical issues faced by the industry. The present study assessed the suitability of non-thermal, high-intensity ultrasound (US) on the inactivation of PPO and POD in fresh Indian Gooseberry juice. Ultrasonic waves, using a 6 mm titanium alloy probe were irradiated in the juice at a maximum power of 455 W and frequency of 20 kHz. The subsequent effects on biochemical attributes were studied using response surface methodology. Inactivation rates of 90.72 % and 73.18 %, respectively, for PPO and POD enzymes, were observed at the highest US intensity and exposure time. Numerical optimisation using the three-factor, three-level Box-Behnken design suggested that an optimum process at 70 % (energy density: 1610 Wcm^−2^) pulsed at 5 s on and 5 s off for 7 min 30 s resulted in PPO and POD inactivation of the order of 76.42 % and 64.57 % respectively. At these experimental conditions, the optimized levels of biochemical attributes i.e., ascorbic acid (738.50 mg/100 mL), total phenols (17.10 mg/mL), DPPH antioxidant activity (58.47 %), tannins (7.11 µg/mL), colour change (Δ*E* = 9.04) and flavonoids (6.14 mg/mL) were achieved. The overall statistical models were significant for all the responses except for reducing sugars. Furthermore, the approximation equations for individual responses indicated that the goodness of fit was adequate (*R*^2^ > 0.90). The results suggested that ultrasound is a suitable processing technique for amla juice stabilisation compared to thermal treatments that result in the loss of quality.

## Highlights

• The suitability of high-intensity ultrasound for the inactivation of enzymes in amla juice was investigated.
• Inactivation rates as high as 90.72% and 73.18% for PPO and POD respectively were observed.
• Optimized process included energy density of 1610 W cm^−2^ pulsed at 5 s on and 5 s off for 7 min 30 s.
• The goodness of fit for approximation of responses was adequate (*R^2^* > 0.90)

## Graphical abstract

**Figure:**
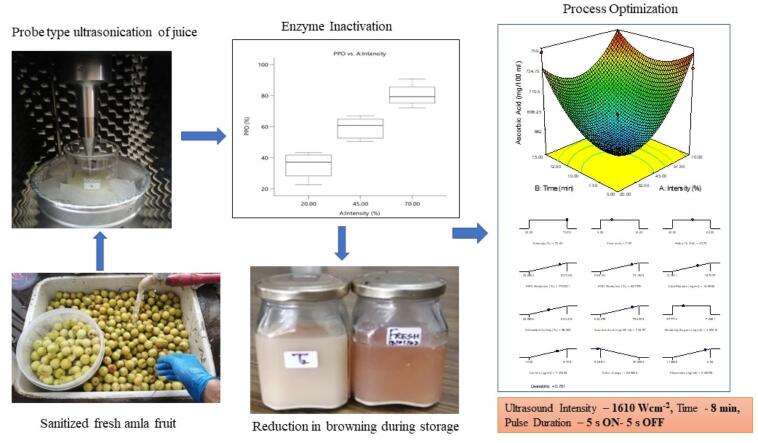

## 1 Introduction

Indian Gooseberry or Amla (*Emblica officinalis*) is a widely distributed plant of Euphorbiaceous in subtropical and tropical areas of India, China, Indonesia, and Malaysia [28]. As an important fruit in the Indian subcontinent, it holds a significant relevance in indigenous medicine. The fruit is a rich natural source of vitamin C and total phenols, antioxidants, flavones, and tannins, among other bioactive compounds [28]. The bio-actives present in amla exhibit hypolipidemic and hypoglycaemic properties and form a major constituent of hepatoprotective formulations [22]. However, its applicability as a table fruit is limited due to its astringent nature, high acidity, poor flavour and low TSS. Moreover, it is highly perishable and has limited storability [26]. The shelf life is usually extended by cold storage, sun drying, convective drying or processing into secondary value-added products like juice syrup, murabba, squash, pickle or dehydrated powder [23]. Reduction in ascorbic acid content and browning of juice due to enzymatic activity are the two main limiting factors in processing amla juice [27]. Enzymes such as peroxidases (POD) and polyphenol oxidase (PPO) are of significant importance as they have been reported to cause quality degradation in fruits and vegetables [31]. In particular, POD is one of the most thermally stable enzymes and is responsible for the catalysis of several redox reactions that cause quality deterioration in plant products [18]. It is also considered an index of success for enzyme inactivation processes in the food industry. It is generally believed that if there is no POD activity, other heat-resistant enzymes like catalase activities need not be evaluated [18]. The activity of PPO, on the other hand, is responsible for the undesirable browning and off-flavours of amla juice and is thus a key constituent in the determination of product acceptance [14].

Conventionally, shelf-stable juice is made with the help of thermal pasteurisation at 75–95 °C or sterilisation at temperatures exceeding 100 °C, followed by the incorporation of certain chemical preservatives [19], [33]. However, this process can adversely affect thermally sensitive components in amla, resulting in loss of quality and browning. There is growing consumer demand for safe and minimally processed foods involving the use of non-thermal technologies that has encouraged the food industry to explore the feasibility of new technologies like ultrasound, pulsed electric field, high hydrostatic pressure, pulsed light, etc. [2]. Ultrasonication is one of the powerful non-thermal techniques that has recently gained considerable importance for its potential in food processing operations due to its effectiveness, simplicity, portability and low cost [4], [29]. The process involves the irradiation of food products with high-energy and low-frequency sound waves (20 kHz − 1 MHz; intensity > 1 W/cm^2^), causing agitation in the propagating medium [8]. While the waves traverse through the medium, they cause alternate compressions and decompressions, which create, expand and implode microbubbles in a process termed ‘acoustic cavitation’. The implosion is associated with releasing high energy (up to 50 MPa; 5000 °C), shock waves and microjets in a short period [4]. In addition, the changes associated with ultrasound processing are brought about by several mechanisms, including agitation, rarefaction, compression, sponge effect, wave distortion, production of free radicals, etc. Several researchers have discussed these process mechanisms in detail [15]. Besides, an extensive review on the application of ultrasound in several food processing operations has been carried out by Aslam et al. [4].

Many studies evaluating the effects of ultrasonication on fruit juices in the literature confirm the suitability of the process in improving the quality of processed fruit juices. However, despite its nutritional and neutraceutical potential, no report extends the suitability of the US process to amla juice. Therefore, a chemometric experimental design using the response surface methodology was established to evaluate the feasibility of probe-type ultrasonication on the enzyme inactivation and retention of total phenolics, flavonoids, reducing sugars, antioxidant activity, ascorbic acid, tannins and color attributes in the treated amla juice.

## 2 Materials and methods

### 2.1 Preparation of amla juice

Fresh Indian gooseberry (*Embilica officinalis* L. var. Neelum) fruits were procured from the fruit farms of Punjab Agricultural University, Ludhiana, India and sorted for uniform size (30 ± 3 mm diameter, fully ripe). The fruits were sanitised using 100 mg/L sodium hypochlorite solution for 10 min, followed by drying using blowing air. The sanitised fruits were frozen in a chest freezer at −20 ± 1 °C for 24 h, followed by thawing at 4 °C for another 10–12 h as a pre-treatment for manual deseeding and segmentation. The freeze–thaw cycle softens the fruit tissue and eases the deseeding process. The fruits were pulped with the help of a household juicer and filtered using a double-layered muslin cloth. Subsequently, the juice was stored in 5 L stainless-steel (3 1 6) containers at 4 °C until further processing.

### 2.2 Ultrasound treatments

**Fig. 1:**
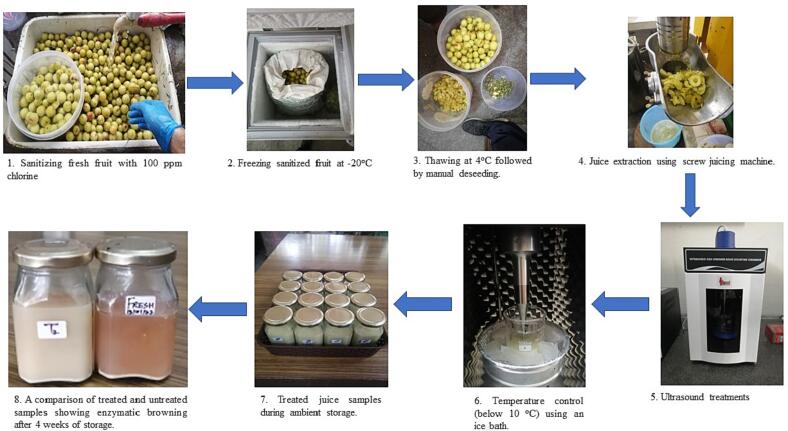
Experimental Setup for ultrasonication of amla juice and the flow of processes.

A 650 W, 20 kHz probe-type ultrasonic processor (PRO-650, Labman Scientific Instruments, Chennai, IN) with a 6 mm (1/4″) diameter titanium alloy probe tip was used for juice treatments (Fig. 1). The temperature inside the treatment vessel was monitored continuously with the help of a thermocouple attached to the system interface. The amplitude control of the sonicator was varied between (20–70 %) to select the desired power level. In addition, the pulse on/off duration and exposure time were varied with the help of the in-built functions of the setup. Corresponding intensity levels (I) from the probe were calculated as per Fonteles et al. [17] (Eq. (1)) at an intensity range of 20–70 % and were 465–1625 W/cm^2,^ respectively.

(1) $$I=\frac{P}{\pi r^{2}}$$

where P is the ultrasound power (W) and r is the tip radius (cm).

Amla juice (200 mL) was treated in a 250 mL glass beaker (diameter 6 cm × height 12 cm) inside an ice bath to restrict the temperature from rising above 10 °C. The probe was immersed up to a depth of 25 mm inside the glass beaker. All the treatments were carried out in triplicates. The samples for enzyme analysis were frozen at −20 ± 1 °C, and those for other biochemical tests were stored at 4 °C until further analysis within 48 h.

### 2.3 Experimental design

After a preliminary review of the literature [4], the process was assumed to be affected by three major variables, viz. ultrasound power intensity (460–1610 W/cm^2^), treatment time including both on and off pulses (5–15 min) and pulse duration (4–6 s/10 s ON) which were controlled and monitored accurately. The experimental plan for the three variables selected was finalised as per the design of Box and Behnken [12] under Response Surface Methodology (RSM) as suggested by a number of researchers [6], [7], [32]. The design was primarily intended to study the inactivation of peroxidase (POD) and polyphenol oxidase (PPO) activities in the treated amla juice. However, the process was also expected to have a profound effect on the quality as well as visual characteristics (responses) of treated juice which were studied in terms of the effects on ascorbic acid (mg/100 mL), total phenolic content (mg/mL), antioxidant activity (DPPH free radical scavenging activity, %), total flavonoids (mg/mL), tannins (ug/mL), reducing sugars (mg/mL) and the observed colour change (Δ*E*). The effect of the process variables selected for the present study and the levels thereof were analysed using the Design Expert DX 14 (Statease, Minneapolis, US). The design summary included a total number of 15 experiments which were done in duplicates, and response values are provided as means. Individual responses were analysed using quadratic models and the numerical optimisation process was aimed at obtaining a combination of variables that could provide the maximum reduction in enzymatic activity and the best retention of biochemical and colour attributes. The constraints for optimization were set according to the intended outcome of US process. The optimization goal for process parameters was selected to be ‘in the range’ while as the goal was set as maximization for all responses except color change. All responses for which there was a non-significant effect of US process were excluded from optimization process. Furthermore, in order to accommodate optimized processing parameters for each individual response (Table 4), a set of numerical solutions aimed to achieve maximum desirability for the target response have also been presented (Fig. 4).

### 2.4 Enzymatic activity

#### 2.4.1 Peroxidase (POD) activity

The peroxidase activity was estimated following Chakraborty et al. [14] with slight modifications. The fresh treated juice was centrifuged in 2 mL Eppendorf tubes at 10,000 × g for 10 min at 4 °C (Remi, Chennai, IN). A half millilitre of the supernatant was added with 1 and 2 mL of 0.2 M guaiacol solution and phosphate buffer (pH 6.5), respectively. The absorbance at 420 nm was observed after an interval of 30 s for a duration of 180 s using a UV–vis spectrophotometer. The corresponding absorbance change was plotted, and the linear portion of the graph was used to estimate the enzymatic activity. The results are expressed in terms of percentage reduction as follows (Eq. (2)):

(2) $$Enzymeactivityreduction\left(\%\right)=\left(\frac{{\mathrm{Abs}}_{\mathrm{fresh}}-{\mathrm{Abs}}_{\mathrm{sample}}}{{\mathrm{Abs}}_{\mathrm{fresh}}}\right)*100$$

where Abs_fresh_ and Abs_sample_ are the corresponding absorbance values of fresh and treatment samples, respectively.

#### 2.4.2 Polyphenol oxidase (PPO) activity

The activity of the polyphenol oxidase enzyme was estimated using the method followed by Saxena, Makroo & Srivastava [39]. Similar to the first steps of POD estimation, the fresh juice was initially centrifuged at 10,000 × g for 10 min at 4 °C. Briefly, 1.5 mL of the supernatant was added to 0.5 mL pyrocatechol (0.5 mol/L) and 3.0 mL potassium phosphate buffer (pH 6.8). The increase in absorbance was noted at 410 nm after every 30 s for a duration of 180 s. Eqn. (2) expresses the results as a percentage reduction of PPO compared to fresh untreated samples.

### 2.5 Measurement of colour attributes

The color attributes of the treated juice were measured using Colour Reader CR-10 (Konica Minolta Sensing Inc, Japan) in terms of the CIE LAB parameters, viz. L* (lightness/darkness), a* (red/green) and b* (blue/yellow). The samples were analysed in a 250 mL glass beaker, and the sensing bulb was so placed that no light could pass through except for the illumination generated by the equipment. The color change was calculated using the following Eq. (3) [5].

(3) $$\Delta E=\sqrt{{{\left(L-L_{0}\right)}^{2}+\left(a-a_{0}\right)}^{2}+{\left(b-b_{0}\right)}^{2}}$$

where L_0_, a_0_ and b_0_ represent the respective readings of fresh amla juice.

### 2.6 Determination of total phenols

Total phenols in the treated amla juice were determined using the Folin–Ciocalteu method described by Waterhouse [47] with some modifications. Briefly, 0.5 mL of treated/fresh amla juice was mixed with 9.5 mL of 80 % methanol and refluxed at 80 °C for 10 min. The mix was then centrifuged at 10,000 g and filtered using a Whatman filter paper to make the final volume of 10 mL with the addition of 80 % methanol. Half millilitre of 20-fold diluted amla juice was mixed with equal volumes of 1:1 diluted Folin–Ciocalteu reagent. The mix was allowed to react for 5 min, after which 1 mL of 35 % Na_2_CO_3_ (w/v) was added. The contents were placed in the dark for one hour, and the absorbance was measured at 765 nm using a UV–vis spectrophotometer. Standard gallic acid dissolved in methanol in different concentrations was used to develop a standard calibration curve. Total phenolic content is expressed as milligrams of gallic acid equivalent per millilitre of amla juice (mg GAE/mL).

### 2.7 Antioxidant capacity

The antioxidant activity of amla juice was measured in terms of its free radical scavenging activity using the 2,2-diphenyl-1-picrylhydrazyl (DPPH) test as per Kaushik., Kaur, Rao & Mishra [24] with some minor modifications. The methanolic extract of 0.5 mL juice was prepared for total phenols determination for the DPPH assay. Briefly, 3.9 mL of freshly prepared DPPH solution (25 mg in 1 L of 80 % methanol) was added to a 10 µL centrifuged aliquot of methanolic extract and 90 µL of double distilled water. The solution was then allowed to stand in the dark for 30 min, and the change in absorbance due to the scavenging of free radicals was measured at 517 nm. The control sample was prepared by adding 10 µL of 80 % methanol in place of juice extract, and the absorbance was used to calculate free radical scavenging activity (FRSA, %) using the following Eq. (4).

(4) %FRSA=[(Ac-At)/Ac]∗100

where A_c_ and A_t_ are the observed absorbance values of control and test samples, respectively.

### 2.8 Ascorbic acid

Ascorbic acid content (mg/100 mL) of amla juice was determined using titration with standardised 2,6-Dichlorophenol-Indophenol dye (DID) solution [36]. Briefly, 0.25 mL of amla juice was added to 10 mL of metaphosphoric acid solution (15 g metaphosphoric acid + 40 mL acetic acid in 450 mL double distilled water). Only 5 mL from this solution was further titrated with the dye (52 mg DID + 42 mg NaHCO_3_) until a pink color appeared.

### 2.9 Reducing sugars

The reducing sugars in amla juice were estimated using the method proposed by Nelson [30] with some alterations. Reagent A was made using sodium carbonate (25 g), sodium bicarbonate (20 g), potassium sodium tartrate (25 g) and sodium sulphate (200 g) in one litre of distilled water. Similarly, reagent B was prepared by dissolving copper sulphate (15 g) in 80 mL of distilled water, followed by 2–3 drops of concentrated sulphuric acid. The coloured dye solution for estimation was prepared separately by adding ammonium molybdate (25 g), sulphuric acid (21 mL) and sodium arsenate (12 % w/v) in 450 mL of distilled water. In order to make the test sample, distilled water was added to 0.2 mL of the juice sample (clarified using lead acetate overnight) to make the final volume of 1 mL. To this, 1 mL of a freshly prepared mixture of reagents A and B (25:1 v/v) was added. The solution was heated in a hot water bath (80 °C) for 10 min. After cooling, 1 mL of ammonium molybdate solution was added, and the change in absorbance was noted at 520 nm. Standard curves were obtained using glucose and xylose as standards (20–100 µg), and the concentrations are expressed in mg/mL.

### 2.10 Total flavonoids

Total flavonoids in amla juice were estimated using the method of Balbaa, Zaki, & El Shamy [9] with some modifications. Briefly, 0.5 mL of methanolic extract was prepared to determine total phenols, evaporated to dry using a hot water bath (80 °C), and then added to 10 mL of 0.1 M methanolic solution of aluminium chloride. The samples were then incubated for 30 min in the dark, and the changes in yellow colour developed were measured at 420 nm against blank using a UV–vis spectrophotometer. The concentrations are expressed as mg/mL using a standard curve obtained using rutin (40–200 μg/ml).

### 2.11 Tannins

Tannins in amla juice were estimated following the method of Price, Hagerman & Butler [34]. To 0.5 mL of juice sample, 20 mL of 1 % HCl in methanol was added. The test tubes containing the mix were placed in a shaking water bath at 20 °C for 20 min. The mix was then centrifuged at 10,000 × g, and the supernatant was used for estimation. Briefly, 5 mL of 0.5 g vanillin dissolved in methanol containing 4 % HCl was added to the supernatant, and the solution was incubated for 20 min. The change in absorbance was noted at 500 nm against 4 % HCl as blank. The standard curve was prepared using catechin (5–200 µg), and the concentrations are expressed in µg/mL.

### 2.12 Statistical analysis

The results are expressed as means or means ± standard deviations. Comparisons between the experiments, as indicated in the design summary, as well as the adequacy of the fitted models, were done using the one-way analysis of variance (ANOVA) using the Design Expert v 14.0 (Statease Inc, Minneapolis, US) software.

## 3 Results and discussion

Ultrasonication of fresh amla juice was conducted by varying three process parameters, viz. ultrasound intensity (20–70 %; 460–1610 W/cm^2^), exposure time (5–15 min) and pulse duration (4–6 s on/10 s) and the process was optimised based on the corresponding effects of the process on selected responses (PPO and POD inactivation rates, ascorbic acid, total phenols, antioxidant activity, reducing sugars, flavonoids, tannins and color change) (Table 1). The effects of process variables on selected responses of amla juice were plotted as three-dimensional response surface graphs (Fig. 3). The analysis of variance for all the responses has been presented in Table 2, the model equations for the approximation of responses in Table 3 and the effects on responses have been discussed as follows.

**Table 1:** Summary of 3 factor, 3 level Box-Behnken design to study the effects of US intensity, exposure time and pulse duration on the selected responses of fresh amla juice.

**Sample ID**	**Intensity (%)**	**Time (min)**	**Pulse (% ON)**	**PPO reduction (%)**	**POD reduction (%)**	**Total phenols (mg/mL)**	**Flavonoids (mg/mL)**	**Antioxidant activity (% DPPH FRSA in 10 µL extract)**	**Ascorbic acid (mg/100 mL)**	**Reducing Sugars (mg/ml)**	**Tannins (µg/mL)**	**Color change (ΔE)**	
												**After 4 weeks**	**AT**
**Fresh**	0.00	0.00	0.00	0.00	0.00	16.73	7.25	52.21	606.90	7.49	1.56	20.71	0.00
T_1_	20.00	10.00	40.00	43.30	33.52	13.48	4.44	56.43	643.68	6.54	2.30	20.07	1.69
T_2_	45.00	10.00	50.00	60.82	39.66	13.38	3.47	57.18	662.07	6.60	8.84	11.04	1.32
T_3_	70.00	10.00	60.00	80.41	60.89	15.22	6.95	59.81	712.64	4.64	5.44	12.81	1.74
T_4_	70.00	5.00	50.00	72.16	70.39	18.70	6.77	57.56	735.63	4.96	5.40	12.34	1.45
T_5_	70.00	10.00	40.00	78.35	59.22	14.68	6.47	61.88	744.83	5.15	4.92	9.66	1.87
T_6_	70.00	15.00	50.00	90.72	73.18	14.66	2.70	62.44	754.02	3.78	5.29	9.83	0.56
T_7_	45.00	10.00	50.00	58.76	41.90	14.92	2.68	56.43	680.46	6.67	9.71	10.36	1.54
T_8_	45.00	5.00	60.00	52.58	36.31	15.87	2.79	57.18	669.43	7.64	4.84	18.22	2.05
T_9_	45.00	15.00	40.00	64.95	43.58	13.08	6.06	57.75	685.06	5.27	2.45	16.21	1.33
T_10_	45.00	5.00	40.00	50.52	34.64	14.08	1.36	60.94	671.26	5.06	1.06	18.37	1.00
T_11_	45.00	15.00	60.00	67.01	48.60	14.83	1.34	60.56	698.85	6.14	3.91	15.31	1.29
T_12_	20.00	5.00	50.00	22.68	26.82	13.62	1.16	56.43	675.86	7.45	6.40	18.22	1.44
T_13_	20.00	15.00	50.00	40.21	31.84	13.36	2.03	56.24	754.02	6.92	2.03	12.54	1.20
T_14_	45.00	10.00	50.00	61.86	38.55	14.55	3.11	54.93	680.46	5.03	7.73	11.83	1.29
T_15_	20.00	10.00	60.00	34.02	31.84	14.05	2.30	53.99	662.07	7.07	5.14	19.40	0.76

FRSA: Free Radical Scavenging Activity; AT: After treatment; values are given as means for n = 3.

**Fig. 2:**
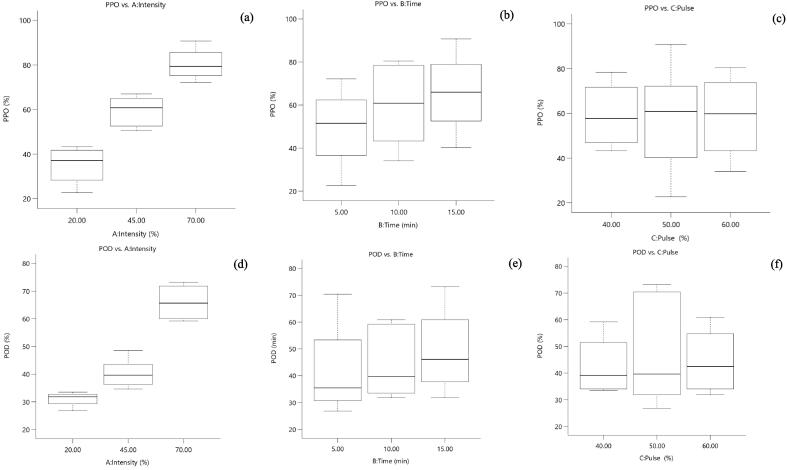
Graphical representations expressing the effects of US process parameters on the inactivation of PPO and POD (Values are mean ± sd, n = 3).

**Fig. 3:**
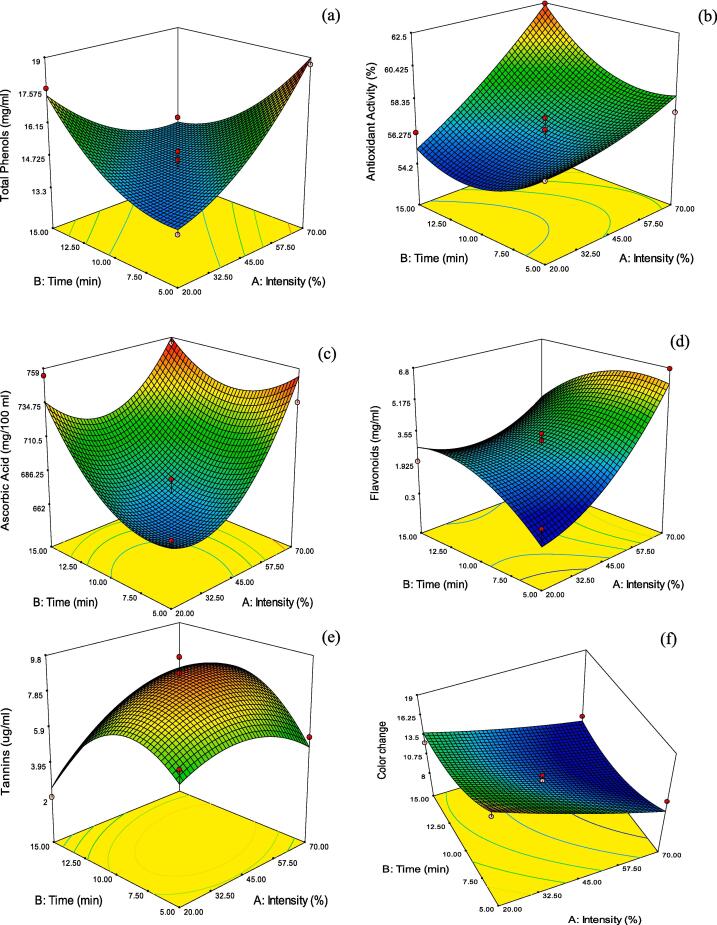
Effects of US intensity and time on (a) total phenols (mg/ml) (b) Antioxidant activity (%) (c) Ascorbic acid (mg/100 mL) (d) Flavonoids (e) Tannins (µg/ml) (f) Color change (ΔE) of sonicated amla juice.

**Fig. 4:**
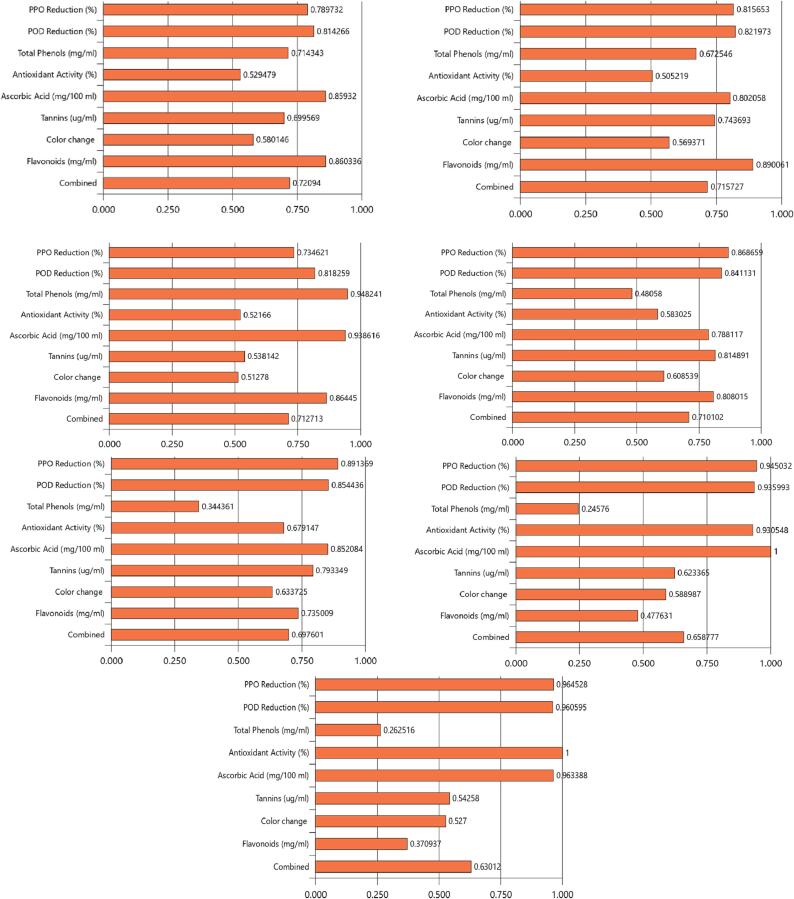
Desirability of optimal solutions for the individual responses of (a) combined overall (b) flavonoids (mg/mL) (c) total phenols (mg/mL) (d) Tannins (µg/mL) (e) color change (ΔE) (f) Ascorbic acid (mg/100 mL) (g) Antioxidant activity, PPO and POD inactivation for ultrasonication of fresh amla juice using the numerical optimization technique**.**

**Table 2:** ANOVA for quality responses of US treated amla juice.

Source	Responses								
	PPO reduction (%)	POD reduction (%)	Total phenols (mg/mL)	Flavonoids (mg/mL)	Antioxidant activity (% DPPH FRSA in 10 µL)	Ascorbic acid (mg/100 mL)	Reducing Sugars (mg/mL)	Tannins (µg/mL)	Color change (*ΔE*)
Model	37.04*	10.65*	6.84	5.04	5.24	5.36*	2.32	8.14	7.95*
A-US Intensity	291.49*	82.56*	4.77	17.90	24.81	16.25*	15.39	3.05	32.31*
B- Time	37.35*	3.57*	0.99	2.57 × 10^−4^	1.71	7.10*	1.56	1.84	8.68*
C-Pulse	0.085	0.19	5.29	2.60	2.13	1.23 × 10^−3^	2.08	8.39	0.10
AB	0.019	0.042	32.05	5.20	3.69	2.59	0.15	4.13	0.99
AC	2.28	0.095	5.13 × 10^−4^	1.46	0.020	1.86	0.37	1.22	1.44
BC	0.000	0.095	9.12 x10^−4^	8.05	6.20	0.18*	1.01	1.22	0.055
A^2^	0.93	8.11	5.69	3.93	0.44	11.41	0.32	5.67	0.14
B^2^	1.21	0.76	7.12	3.39	4.99	5.65	0.029	24.10	5.00
*C*^2^	0.048	0.37	4.77	2.22	4.11	2.79	3.93 × 10^−5^	30.46	24.51*
Lack of Fit	8.82	16.23	0.66	11.68	1.54	4.42	0.74	1.21	7.12
C.V. (%)	6.42	12.15	4.82	30.27	2.28	2.67	14.36	20.86	11.04

*represents that the quantity is significant (*p* < 0.05); AB, AC and BC represent the interaction terms of process parameters and C.V. is the coefficient of variation.

**Table 3:** Second order model equations for the approximation of selected responses of amla juice treated by subjecting to ultrasonication.

Parameter	Model Equation	R^2^
PPO reduction (%)	$\mathrm{PPO}=27.82+\left(0.59xA\right)+\left(3.25xB\right)-\left(0.98xC\right)+\left(0.01xAC\right)-\left(0.09xB^{2}\right)$	0.99
POD reduction (%)	$\mathrm{POD}=3.97-\left(0.58xA\right)-\left(1.88xB\right)+\left(1.49xC\right)+\left(0.017xBC\right)+\left(0.013xA^{2}\right)+\left(0.099xB^{2}\right)-\left(0.017xC^{2}\right)$	0.95
Total phenols (mg/mL)	$\mathrm{TP}=-10.09+\left(0.06xA\right)-\left(0.11xB\right)+\left(0.88xC\right)-\left(0.02xAB\right)+\left(0.04{xB}^{2}\right)$	0.92
Flavonoid Content (mg/mL)	$\mathrm{FC}=9.80-\left(0.13xA\right)+\left(2.81xB\right)-\left(0.71xC\right)-\left(0.03xBC\right)-\left(0.04xB^{2}\right)$	0.90
Antioxidant activity (% DPPH FRSA in 10 µL extract)	$\mathrm{FRSA}=118.45-\left(0.09xA\right)-\left(3.2xB\right)-\left(1.81xC\right)+\left(0.01xAB\right)+\left(0.03xBC\right)+\left(0.06xB^{2}\right)+\left(0.01xC^{2}\right)$	0.90
Ascorbic acid (mg/100 mL)	$\mathrm{AA}=258.63+\left(0.09xA\right)-\left(13.39xB\right)+\left(17.6xC\right)-\left(0.12xAB\right)-\left(0.05xAC\right)+\left(0.08xBC\right)+\left(0.05xA^{2}\right)+\left(0.92xB^{2}\right)-\left(0.16xC^{2}\right)$	0.91
Reducing Sugars (mg/mL)	$\mathrm{RS}=-1.58+\left(0.05xA\right)+\left(0.47xB\right)+\left(0.18xC\right)$	0.81
Tannins (µg/mL)	$\mathrm{Tannins}=-94.25+\left(0.24xA\right)+\left(2.24xB\right)+\left(3.34xC\right)-\left(0.011xBC\right)-\left(0.107xB^{2}\right)-\left(0.03xC^{2}\right)$	0.94
Color change (ΔE)	$\mathrm{CC}=139.77-\left(0.43xA\right)-\left(1.91xB\right)-\left(4.22xC\right)+\left(0.07xB^{2}\right)+\left(0.04xC^{2}\right)$	0.93

A, B and C are process parameters where A is US intensity (%), B is time (min) and C is pulse duration (ON%).

**Table 4:** A list of the optimized solutions based on the prediction of the most feasible values of individual response attributes.

Intensity (%)	Time (min)	Pulse (% ON)	PPO Reduction (%)	POD Reduction (%)	Total Phenols (mg/mL)	Antioxidant Activity (%)	Ascorbic Acid (mg/100 mL)	Tannins (ug/mL)	Color change	Flavonoids (mg/mL)	Desirability
70.00	7.49	49.58	76.42	64.57	17.10	58.47	738.50	7.11	9.04	6.14	**0.72**
70.00	8.03	52.10	78.18	64.93	16.86	58.26	732.18	7.49	9.25	**6.31**	0.72
70.00	5.74	50.38	72.67	64.76	**18.41**	58.40	747.25	5.72	10.32	6.17	0.71
70.00	10.04	51.76	81.79	65.82	15.78	58.92	730.64	**8.11**	8.50	5.84	0.71
70.00	11.55	48.59	83.33	66.43	15.02	59.73	737.70	7.92	**8.02**	5.42	0.70
70.00	14.49	49.68	86.98	70.22	14.46	61.85	**754.43**	6.45	8.87	3.93	0.66
70.00	14.72	53.99	**88.31**	**71.36**	14.56	**62.70**	749.98	5.75	10.05	3.31	0.63

### 3.1 Effect of US treatments on enzyme inactivation

#### 3.1.1 PPO inactivation

Polyphenol oxidase is a copper-containing enzyme that has been regarded as the most significant factor for enzymatic browning in fresh fruit juices [31]. US at the selected intensities had a significant effect (*p* < 0.05) on the inactivation of PPO in amla juice. The present study observed that the US intensity and treatment time significantly affected the amla PPO inactivation, while pulse duration had a non-significant effect. The overall model equations were quadratic and accounted for around 98 % of the variations in PPO inactivation during the US treatments (Table 3). It was observed that the reduction (%) in the activity of PPO in amla juice increased as the US intensity and treatment time increased, as illustrated in Fig. 2. The PPO activity decreased in the range of 22.68 % to 90.72 %, depending on the magnitude of the parameters selected. The highest reduction was observed in the case of US treatment performed at 1610 W/cm^2^ US energy density, 15 min treatment time and a pulse duration of 5 s on – 5 s off, while the lowest reduction was observed in the treatment involving US intensity of 460 W/cm^2^, 5 min treatment and a pulse duration of 5 s on – 5 s off. The inactivation tendency of amla juice PPO as affected by different process variables of US treatments is shown in Fig. 2. It can be observed that the PPO inactivation rates increased dramatically when the intensity was increased. The vibration energy produced during US treatments causes cell lysis and subsequently inactivates enzymes by generating cavitation bubbles and short-lived spots of extremely high pressure and temperature upon implosion [38]. Many studies in the literature confirm these findings. However, in many cases, the inactivation rates vary depending on the experimental conditions, nature of fruit juice treated, temperature control, etc. In a recent study, Cao, Cai, Wang & Zheng [13] reported similar PPO reduction percentages in bayberry juice. The authors observed that it was possible to cause as high as 97 % inactivation in the PPO activity in the juice at an ultrasound energy intensity of 452 W/cm^2^ exposed for 8 min with proper temperature control below 10 °C. In addition, complete inactivation was reported in the same study when the US was combined with heat. Although PPO is the first factor of enzymatic browning, it is not thermally stable and can be inactivated by exposure to high temperatures up to 80 °C for a few minutes [31]. This can be a possible explanation for a higher rate of its inactivation during the present experiment. On the other hand, few reports show a relatively lower rate of PPO inactivation in the case of apple juice [42], where the authors observed a maximum reduction of 57 %, possibly due to the differences in the experimental setup and ultrasound energy density. Sulaiman, Soo, Farid & Silva [43] indicated that the difference in PPO inactivation in fruit purees also depends upon the nature of the fruit and pear PPO was more resistant, followed by apple PPO.

#### 3.1.2 POD inactivation

Peroxidase (POD) is a heat-stable, heme-containing enzyme used to evaluate the efficiency of blanching operations in fruits and vegetables [31]. It is a quality inhibiting enzyme responsible for developing off-flavours and browning pigments in fresh fruits and vegetables. US significantly decreased the POD activity in amla juice at the intensities studied. Of all the parameters studied, US intensity and treatment time had a remarkable effect (p < 0.05), while pulse duration non-significantly influenced the inactivation of amla POD. The model accounted for 95 % of the total variations and adequately expressed the effect of process variables on the POD inactivation rate. The inactivation trends were similar to those observed for PPO and increased with an increase in intensity and treatment time. However, the reduction in POD was comparably lower than PPO. The maximum reduction (73.18 %) was observed for an energy density of 1610 W/cm^2^ at a pulse duration of 5 s on – 5 s off and exposed to the juice sample for 15 min, while the minimum reduction (26.82 %) was reported for the juice samples sonicated at 460 Wcm^−2^, pulse duration of 5 s on – 5 s off and a treatment time of 5 min. The central experiment (1035 Wcm^2^, 5 s on – 5 s off, 10 min) illustrated a reduction in POD content up to 41.9 %. The inactivation potential of amla juice POD as affected by different ultrasound process variables is presented in Fig. 2. In the present study, POD showed higher resistance to inactivation than PPO, probably due to its stable chemical structure, making it resistant to thermal treatments. Similar findings have been reported during sonication of tomato puree, in which the authors observed a POD reduction of up to 83.79 % at 40 % (23 kHz power; 150 s) [16]. Similarly, Saeeduddin et al. [37] reported POD inactivation of around 43 % during thermo-sonication of pear juice (20 kHz; 750 W; 10 min). In a recent study, POD activity in bayberry juice during cold ultrasonication was reduced by up to 90 % using 452 W/cm^2^ US for an exposure time of 8 min [13].

### 3.2 Effect of US on the quality attributes of amla juice

#### 3.2.1 Changes in total phenol content (TPC), flavonoids and reducing sugars

The biochemical compounds in fruit juices are considered very beneficial to human health due to their potential role in controlling or preventing various diseases in the human body [1]. In this context, amla juice is rich in phenolic compounds and is important in contemporary medicine in the Indian subcontinent. Fresh amla juice had a total phenolic content of 16.73 mg GAE/mL, and the effects of various ultrasonication treatments are presented in Fig. 3a. The US treatments at the configurations studied in the present work reduced the total phenolic content compared to the control depending on the intensity and treatment time. The effect of intensity on the attribute was significant (*p* < 0.05), while exposure time and pulse non-significantly contributed to the variations (Fig. 3a). However, the significance of US intensity was sufficient to make the overall model adequate (*p* < 0.05). Similar results were observed for flavonoids, wherein the content decreased with US treatments compared to fresh samples (7.25 mg/mL). Among the process variables, the effect of US intensity was most profound, indicating that the variations were more dependent on the US-induced effects, including extractability and oxidative degradation. The total flavonoid content was observed to be highest (6.95 mg/mL) in the samples exposed to 1610 Wcm^−2^ pulsed at 6 s on – 4 s off for 10 min, while the lowest content (1.16 mg/mL) was reported for 460 W cm^−2^ pulsed at 5 s on – 5 s off for 5 min (Fig. 3d). There have been varied results reported about the effects of US treatments on the biochemical attributes in fruit juices, including total phenols and flavonoids. Silva, Arruda, Pastore, Meireles & Saldana [41] observed that the TPC reduced significantly as the US process intensified during high-intensity ultrasonication of orange juice. The authors reported around 26 % decline in TPC after sonication of 1200 W in 10 mL of juice for 10 min duration. These results are in agreement with those observed by Sun, Zhong, Cao, Lin & Ye [44] for apple juice, Fonteles et al. [17] for cantaloupe melon juice, Silva et al. [41] for orange juice and Golmohamadi, Möller, Powers & Nindo [21] for red raspberry puree. During the ultrasonication process, the changes in TPC can be associated with two phenomena, viz., the release of phenolics from the dissociated cellular structures as initiated by increasing US intensity and a subsequent degradation due to the free radicals produced as a result of the US process [45]. It can be observed from the three-dimensional response surface plots (Fig. 3a) that total phenolic degradation is highest at the central experiment, including a 45 % US intensity, indicating that the US-induced degradation is compensated by the release of bound phenolics beyond this point in the plot.

In this study, the reducing sugars in treated amla juice showed a decreasing trend compared to fresh juice (7.49 mg/mL). Among the treatments, the values varied between 3.78 mg/mL (70 % US intensity, 15 min exposure, 5 s pulse on) and 7.64 mg/mL (45 % US intensity, 5 min exposure, 6 s pulse on) with the decrease associated to a subsequent increase in the US intensity. The effect was, however, non-significant (*p* < 0.05) among the studied experimental conditions. Hence, reducing sugars were not used as a criterion for the numerical optimisation of process variables.

#### 3.2.2 Antioxidant activity (DPPH radical scavenging)

The antioxidant activity of amla juice treated with the selected levels of process variables increased compared to non-sonicated samples during the present study. The free radical scavenging activity of 10 µL methanolic extract of amla juice samples on 2,2-diphenyl-1-picrylhydrazyl varied between 53.99 % and 62.44 % among the treatments. In comparison, the fresh samples recorded an activity of 52.21 %, significantly lower than all the treatments. Among the process variables, the effect of US intensity was the most significant contributor to the overall model and accounted for 90 % of the total variations. The increase in the antioxidant activity may be associated with the respective increase in the ascorbic acid concentrations due to the cavitation induced during the US process. The results are in agreement with those observed in apple juice [1], purple cactus pear [49], Kasturi lime juice [11], fresh-cut pineapple [48] and orange juice [25].

#### 3.2.3 Ascorbic acid

Ascorbic acid contributes to antioxidant properties and helps prevent various health issues, including cancer [40]. Indian Gooseberry is one of the richest sources of ascorbic acid; hence, its retention is the primary focus during fresh fruit processing. The results indicated a significant increase (*p* < 0.05) in the ascorbic acid content of sonicated amla juice compared to fresh non-sonicated samples. Among the treatments, the highest concentration of vitamin C (754.02 mg/100 mL) was observed in the juice samples sonicated at 1610 Wcm^−2^ pulsed at 5 s on − 5 s off for 15 min, while the lowest concentration (643.68 mg/100 mL) was observed for experimental conditions of 460 Wcm^−2^, 10 min and 4 s on – 6 s off. It appears that the release of bound ascorbic acid from within the cellular structures had a predominant effect compared to the oxidative degradation in sonicated amla juice. A possible reason for the observation would be the relatively higher stability of ascorbic acid towards reactive oxygen species (ROS) as compared to gallic acid (primary contributor of total phenolics) [10]. In addition, ascorbic acid has been observed to react slowly to ROS compared to gallic acid. Its solubility in water is greater than that of phenolic compounds that might have further assisted in the US-induced extractability [35])**.** Ascorbic acid is highly sensitive to heat and light, wherein the kinetics of degradation follows first-order reactions through aerobic or anaerobic pathways [46]. However, during ultrasound processing, the fruit juice is exposed to low temperatures; the process may have further contributed to the removal of dissolved oxygen from the juice because of cavitation [1]. Similar results have been observed in the case of Kasturi lime juice [11], apple juice [1], purple cactus pear [49], etc. Thus, ultrasound processing could enhance the ascorbic acid content of fresh amla juice.

#### 3.2.4 Tannins

A significant effect (*p* < 0.05) of US process variables was observed in the case of tannins, wherein the content increased compared to the fresh non-sonicated samples (1.56 µg/mL), and the increase was most profound in the central experiment (45 % intensity, 10 min exposure, 5 s pulse on) witnessing total tannin content of 9.708 µg/mL, which was almost six times higher than the fresh samples (1.56 µg/mL). At the highest intensity (70 %) and longest exposure time (15 min), the tannin content of 5.29 µg/mL was observed, indicating the effects of oxidative degradation as a result of cavitation. The approximation model for the prediction of tannins in the US-treated amla juice is presented in Table 3, which accounted for almost 94 % of the total variations during the process. Tannins are secondary phenolic compounds and are classified into hydrolysable and condensed tannins. The hydrolysable tannins are derivatives of gallic acid which occur in fruits as glycosides and are readily water soluble. On the other hand, condensed tannins are by-products of flavonoids and occur as soluble oligomers inside the cell walls [20]. The significant increase in tannins during the present study could have been due to the release of condensed tannins from the cell walls due to ultrasonication-induced cavitation and subsequent conversion of phenolic and flavonoid by-products. A similar trend was observed by Annegowda, Anwar, Mordix, Mordi, Ramanathan, and Mansor [3] while sonicating *T. catappa* leaves. The authors observed a decrease in total phenolic and flavonoid content with a subsequent increase in the total tannin content for leaves sonicated for 20 min exposure time.

#### 3.2.5 Colour attributes

Colour is an important sensory attribute which affects the consumer acceptability and likeness of amla juice. The present study recorded the colour change (Δ*E*) values weekly after the US processing. The values used for numerical optimisation were the Δ*E* values after four weeks of ambient storage (20 ± 6 °C) when there was a significant difference among the treatments. It can be observed that the lowest color change values were recorded by the samples that were subjected to the highest intensity of ultrasound (1610 W cm^−2^) for 10 (ΔE = 9.66) and 15 min (Δ*E* = 9.83). The highest shift in the color attributes was experienced for fresh non-sonicated juice samples (Δ*E* = 20.71). The decreasing trend in the ΔE values followed the PPO inactivation results, highlighting that the browning is associated with increased enzymatic activity. At higher intensities, PPO inactivation was more severe, and as such, the relative inactivation of the enzyme assisted the treated samples in retaining color better. The color attributes recorded immediately after the experiment are also presented in Table 1. An increase in ‘*a*’ and ‘*b*’ values compared to fresh juice could be observed after the treatments. This might be due to the physical, chemical and biological effects associated with cavitation [13]. These effects include accelerated breakdown of susceptible bioactive compounds like enzymes and phenols or isomerisation of carotenoids. However, these ΔE values were less than 2.00, and the changes were not noticeable to the naked eye. These results agree with Cao et al. [13] and Abid et al. [1].

### 3.3 Process optimisation

The numerical optimisation technique using the 3-factor Box and Behnken design (1960) suggested that the optimised ultrasonication process in amla juice can be carried out at an ultrasonic intensity of 70 % (corresponding to an energy density of 1610 Wcm^−2^) for an exposure time of 7 min 30 s and a pulse of around 50 % (5 s on – 5 s off) with overall desirability of 0.72. Based on each attribute, a set of optimised conditions was obtained. These optimised levels are approximated to yield the most favourable quality responses, as expressed in Table 4. The optimised experiment having the maximum desirability was repeated for validation, and the responses were found within a 20 % level of variation, suggesting that the model adequately approximates the ultrasonication process in amla juice. Furthermore, a set of numerical optimisation solutions were obtained based on the possible values of individual response attributes and are presented in Table 4.

## 4 Conclusions

Ultrasound is an emerging non-thermal method that has high relevance in fresh fruit juice processing. The suitability of the ultrasonication process on the inactivation of quality degrading enzymes, including PPO and POD, along with its effects on the biochemical and colour attributes of fresh amla juice, was evaluated. We observed that ultrasound at the power levels selected effectively inactivated the PPO and POD enzymes, with the ultrasonic intensity being the most significant factor. Phenolic compounds, flavonoids and reducing sugars were lower than in fresh juice. At the same time, ascorbic acid, antioxidant activity, colour attributes and tannins were retained better than the fresh untreated juice. The present study's optimisation results recommend using ultrasound at 70 % (1610 W cm^−2^) pulsed at 5 s on and 5 s off for an exposure time of 8 min to achieve possible results for enzyme inactivation in addition to the retention of biochemical quality. After four weeks of ambient storage, the ultrasound-treated juice samples exhibited a lower magnitude of colour change, confirming the suitability of this novel processing method. Based on the results obtained in the present study, ultrasound holds significant potential in the non-thermal processing of high-quality amla juice, subject to further experimentation and confirmation of its suitability at an industrial scale.

## Funding

The first author was financially assisted through a student fellowship from the Indian Council of Agricultural Research, Senior Research Fellowship programme. The second author has received financial support for the work by the ICAR-All India Coordinated Research Project on Post Harvest Engineering and Technology.

## CRediT authorship contribution statement

**Raouf Aslam:** Conceptualization, Methodology, Software, Writing – original draft, Writing – review & editing. **Mohammed Shafiq Alam:** Conceptualization, Resources, Supervision, Writing – review & editing, Funding acquisition. **Asgar Ali:** Validation, Writing – review & editing. **Yang Tao:** Writing – review & editing. **Sivakumar Manickam:** Writing – review & editing.

## Declaration of Competing Interest

The authors declare that they have no known competing financial interests or personal relationships that could have appeared to influence the work reported in this paper.

## Data Availability

Data will be made available on request.
